# Toxicology in the Fast Lane: Application of High-Throughput Bioassays to Detect Modulation of Key Enzymes and Receptors

**DOI:** 10.1289/ehp.0900834

**Published:** 2009-07-31

**Authors:** Christophe Morisseau, Oleg Merzlikin, Amy Lin, Guochun He, Wei Feng, Isela Padilla, Michael S. Denison, Isaac N. Pessah, Bruce D. Hammock

**Affiliations:** 1 Department of Entomology and Cancer Center and; 2 Department of Environmental Toxicology, University of California at Davis, Davis, California, USA; 3 Department of Veterinary Medicine Molecular Biosciences, School of Veterinary Medicine, University of California at Davis, Davis, California, USA

**Keywords:** bioassays, biomarkers, enzyme inhibition, high-throughput assays, triclocarban, triclosan

## Abstract

**Background:**

Legislation at state, federal, and international levels is requiring rapid evaluation of the toxicity of numerous chemicals. Whole-animal toxicologic studies cannot yield the necessary throughput in a cost-effective fashion, leading to a critical need for a faster and more cost-effective toxicologic evaluation of xenobiotics.

**Objectives:**

We tested whether mechanistically based screening assays can rapidly provide information on the potential for compounds to affect key enzymes and receptor targets, thus identifying those compounds requiring further in-depth analysis.

**Methods:**

A library of 176 synthetic chemicals was prepared and examined in a high-throughput screening (HTS) manner using nine enzyme-based and five receptor-based bioassays.

**Results:**

All the assays have high *Z*′ values, indicating good discrimination among compounds in a reliable fashion, and thus are suitable for HTS assays. On average, three positive hits were obtained per assay. Although we identified compounds that were previously shown to inhibit a particular enzyme class or receptor, we surprisingly discovered that triclosan, a microbiocide present in personal care products, inhibits carboxylesterases and that dichlone, a fungicide, strongly inhibits the ryanodine receptors.

**Conclusions:**

Considering the need to rapidly screen tens of thousands of anthropogenic compounds, our study shows the feasibility of using combined HTS assays as a novel approach toward obtaining toxicologic data on numerous biological end points. The HTS assay approach is very useful to quickly identify potentially hazardous compounds and to prioritize them for further in-depth studies.

Although pharmaceuticals and pesticides are evaluated for toxicity at great cost, numerous anthropogenic compounds produced in sizable amounts and present in our everyday environment have not been tested for any toxicologic activity. The recent California Green Chemistry Report (California Department of Toxic Substances Control 2008) illustrates that far more chemicals are in common use than the ones tested for toxicity, and in most cases, there are few or no toxicity data for a large number of these chemicals. Novel international legislation, such as the Registration, Evaluation, Authorization and Restriction of Chemicals (REACH) program implemented in 2007 by the European Union (European Chemicals Agency 2007), requires that all chemicals used in the European Union at more than 1 metric ton/year/company be evaluated for their toxicity over the next decade. Ultimately, the European Union may develop an authorization system to control substances of very high concern and progressively replace them with suitable alternatives where economically and technically viable, unless there is an overall benefit for society of using the substance. The U.S. Environmental Protection Agency has several voluntary programs, including the High Production Volume Challenge Program (U.S. Environmental Protection Agency 1998), that allow compiling of chemical toxicity and hazard information for selected chemicals. It is very likely that additional national and international legislation will be enacted that will require generation of toxicity data for most of the chemicals produced in sizable quantity.

For almost 200 years, laboratory animal testing has been the major tool of toxicologists (Gad 2006). However, such tests have the disadvantages of being both time-consuming and very costly because they require use of large number of animals, and they are not always predictive of human risk. For the implementation of REACH, Scialli (2008) estimated that tens of million of animals will be used at a cost of several hundred thousand dollars per compound, making it very challenging to use experimental animals to complete analysis of the toxicologic effects of many chemicals in a reasonable time frame. Accordingly, there is a need for accurate toxicologic evaluation of xenobiotics to be faster and more cost-effective. Progress in molecular biology, biotechnology, and other fields have paved the way for toxicity testing to be quicker, less expensive, and more directly relevant to human exposures (Gibb 2008). Although it is certain that *in vitro* assays cannot yet replace animal testing (Tingle and Helsby 2006), they may provide essential information that can prioritize and dramatically reduce the use of animal testing assays (Silliman and Wang 2006). However, when considering the prospect of screening tens of thousands of chemicals against hundreds of *in vitro* assays, several important questions need to be answered. Can enzyme- or cell-based bioassays yield useful toxicologic information? Furthermore, can these assays be conducted in a high-throughput and reliable fashion, allowing the rapid screening of thousands of compounds for biological and toxicologic activities?

As part of the University of California–Davis Superfund Basic Research Program, whose aim is to identify biomarkers of exposure and effects of toxic substances, we have developed a library of techniques, including numerous enzyme- and cell-based screening assays (Ahn et al. 2008; Garrison et al. 1996; Han et al. 2004; Huang et al. 2007; Jones et al. 2005; Nagy et al. 2002; Rogers and Denison 2000; Shan and Hammock 2001). Although such assays are routinely used to find novel small chemical inhibitors in the pharmaceutical industry, we tested whether such mechanistically based screening assays can be used to rapidly provide information on the potential for compounds to produce specific biological toxic effects that would identify those requiring further in-depth study. More specifically, we tested whether these assays could be adapted for high-throughput screening (HTS). We selected a small (176 compounds) and structurally very diverse library from among commonly encountered environmental chemicals. We report the results of screening this library with nine enzyme-based and five receptor-based bioassays. These assays were selected because the proteins involved were shown to interact with xenobiotics, and because the *in vitro* effects of these xenobiotics could be related to the *in vivo* activity of these proteins and health effects.

## Materials and Methods

A more detailed account of the materials and methods used is given in the Supplemental Materials, (doi:10.1289/ehp.0900834.S1 via http://dx.doi.org/).

### Chemicals

Most chemicals used in the library were from commercial sources. Chemicals were at least 95% pure and used without further purification.

### Environmental chemicals library

The library was prepared in 2-mL deep-well polypropylene 96-well assay plates. Every compound was dissolved at 10 mM in dimethyl sulfoxide (DMSO). Only compounds totally soluble at 10 mM in DMSO were included in the library. In each plate, the wells in the first column contained only DMSO to serve as controls. In the remainder of the plate, we dispensed one compound per well, with 88 compounds total per plate. We created two plates for a total of 176 compounds. A detailed description of the chemical contents in each plate is presented in the Supplemental Materials, Tables 1 and 2 (doi:10.1289/ehp.0900834.S1). The sealed plates were stored at −20°C until use. Upon use, the plates were diluted to the appropriate concentration using a robotic pipetting station.

### Enzyme preparations

Recombinant human soluble epoxide hydrolase (sEH) was produced in a baculovirus expression system (Beetham et al. 1993) and purified by affinity chromatography (Wixtrom et al. 1988). Recombinant human carboxylesterases CES1, CES2, and CES3; fatty acid amide hydrolase (FAAH); and paraoxonase 2 (PON2) were expressed in baculovirus-insect cells as previously described (Huang et al. 2007; Nishi et al. 2006). The CESs were partially purified as previously described (Nishi et al. 2006), whereas microsomal preparations were used for FAAH and PON2 (Huang et al. 2007). Human liver cytosol and microsome extracts were obtained from BD Biosciences (San Jose, CA). Protein concentration was quantified using the Pierce BCA (bicinchoninic acid) assay (Pierce, Rockford, IL) using bovine serum albumin (BSA) as the calibrating standard.

### Enzyme assays

Although the conditions for each enzyme assay were different (for details, see Table 1), the enzymatic assays were all run in a similar format. Enzymes were used at a concentration that results in linear generation of product with increasing time and protein concentration, as well as yielding a signal that was 3–20 times greater than the background. BSA (0.1 mg/mL final concentration) was added to all buffers just before use to reduce nonspecific inhibition (McGovern et al. 2002). For glutathione *S*-transferase (GST) activities, the buffer was supplemented with 5 mM glutathione. For all the enzyme assays, we tested the compounds at final concentrations of 0.1 and 1 μM.

### Kinetic assay conditions

The dissociation constant of triclosan for CES1 was determined following the method described by Dixon (1972) for competitive tight binding inhibitors, using cyano(6-methoxy-2-naphthyl)methyl acetate (CMNA) as the substrate (Shan and Hammock 2001). Inhibitor concentrations between 0 and 1,000 nM were incubated in triplicate for 5 min in sodium phosphate buffer (pH 7.4) at 30°C with 200 μL of the enzyme solution. Substrate at a final concentration of 5–100 μM was then added. Velocity of the reaction was measured as described above. For each substrate concentration, plots of velocity as a function of inhibitor concentration allow the determination of an apparent inhibition constant (K_Iapp_). The plot of K_Iapp_ as a function of the substrate concentration allows the determination of K_I_ when the substrate concentration is zero. Results were expressed as the mean ± SD of three separate K_I_ measurements.

### Cell-based bioassays

Table 2 presents an overview of the different cell-based bioassays used. For all test compounds, agonist activity in the aryl hydrocarbon receptor (AhR), androgen receptor (AR), and estrogen receptor (ER) assays was determined in the AhR, AR, and ER CALUX (chemically activated luciferase expression) bioassays, respectively. All three CALUX bioassays make use of different cell lines (H1L6.1c2, T47D-AR–positive, and BG1Luc4E2/ER-α–positive, respectively) that contain a stably transfected luciferase gene under the transcriptional control of DNA response elements for the activated AhR, AR, and ER, respectively (Garrison et al. 1996; Han et al. 2004; Rogers and Denison 2000). Activation of the receptor signaling pathway was determined by quantifying the luciferase activity in the absence or presence of a known agonist [2,3,7,8-tetrachlorodibenzo-*p*-dioxin (TCDD), 17β-estradiol (E_2_), or dihydrotestosterone (DHT)]. Results were expressed relative to luciferase activity maximally induced by a reference compound (1 nM TCDD for AhR, 10 nM DHT for AR, 1 nM E_2_ for ER). For these assays, the primary screening of the library was done at 10 μM. Membranes enriched in ryanodine receptors (RyRs) were obtained either from adult rabbit skeletal muscle, a pure type 1 ryanodine receptor (RyR1) source (Saito et al. 1984), or from cardiac ventricular tissue, a pure type 2 ryanodine receptor (RyR2) source (Pessah et al. 1990). Activation or inhibition of the receptors was measured by quantifying the ability of the tested compound at 5 μM to enhance or inhibit the basal binding of [^3^H]Ry (2 nM) in the presence of 20 μM CaCl_2_. After a 3-hr incubation at 37°C, the reactions were quenched by filtration through GF/B-grade glass fiber filters and washed twice with ice-cold harvest buffer containing 20 μM CaCl_2_. [^3^H]Ry binding was quantified by measuring the radioactivity collected on the filter.

### Selection of positive hits and counterscreening

For the enzyme assays, a compound was selected as a positive hit if it resulted in > 50% inhibition at the lower concentration (100 nM) and if it resulted in more than 60% inhibition at the higher concentration (1 μM). For the cell-based assays, we selected compounds that significantly (*t*-test and *F*-test, *p* < 0.01) induced the receptor activation of gene expression. For counterscreening, fresh solutions of all positive compounds were prepared in DMSO. For the enzyme assays, the concentration of each compound that inhibited 50% of the enzyme activity (IC_50_) was determined by measuring enzyme activities in the absence and presence of increasing concentrations of inhibitor (ranging from 0.5 to 10,000 nM). IC_50_ values were calculated by nonlinear regression of at least five data points using SigmaPlot, version 9.01 (Systat Software Inc., Chicago, IL). Results are provided as the mean ± SD of at least three separate measurements. Similarly, half-maximal effective concentration (EC_50_) values for agonists of the AhR and ER bioassays were determined, and the results are presented as the mean of triplicate analysis. For the assay of [^3^H]Ry binding to RyR1 or RyR2, the influence of 5 μM of each compound was screened for its ability to either enhance or inhibit specific radioligand binding more than twice the baseline (defined as the level of [^3^H] Ry-specific binding in the presence of DMSO alone). Therefore, a positive hit on RyR1 or RyR2 was defined as ≥ 200% of control binding for activators, or ≤ 50% of control for inhibitors.

## Results and Discussion

### Assays characteristics and positive hits selection

Using results from the blank and full activity controls, we evaluated the suitability of each assay for use as HTS assays. We therefore calculated the signal-to-background ratio (S/B), the signal-to-noise ratio (S/N), and the *Z*′ factor as defined by Zhang et al. (1999). As shown in Table 3, we found that S/B ratios varied from 2.5 to > 150, with the lowest value for the absorbance-based assay (GSTs) and the highest for the radioactive-based assays (RyRs). Similarly, the S/N ratios varied greatly, with a lower value for the absorbance assay and the higher values for the radioactive-based assays. In general, the enzyme-based assays yielded higher *Z*′ factors than did the cell-based bioassays. For the enzyme assays, *Z*′ values were > 0.7, indicating very good and reliable assays that are easily suitable for HTS assays. Although the cell-based assays yielded lower *Z*′ factors, the values were still > 0.5, suggesting that the discrimination is adequate and that these assays could be used in HTS assays. Nevertheless, for the RyR assays, a larger separation band and higher *Z*′ factor could be obtained by reducing the SD of the signal, which was around 20%.

The aims of the primary screening were to identify all possible positive hits and to ensure there were no false negatives. Thus, for the primary screening of the library, we tested the xenobiotics at relative high concentrations (0.1 and 1 μM for the enzymes, and 5 and 10 μM for the receptors), which should be far higher than blood concentrations resulting from exposure. Thus, it is unlikely that compounds found negative in the primary screening will be false negative and affect the tested proteins *in vivo*. Generally, testing higher concentrations result in solubility problems for an increasing proportion of compounds. Based on our definition of positive hits (described above), for the 14 assays we obtained a total of 69 positive results (Table 3), which represent on average five positive hits per assay, or 3% of the library. For FAAH, GST, and AR bioassays, we obtained no hits from the screening. There were twice as many positive hits from chemicals in plate II (42) than from those in plate I (27) [see Supplemental Material, Figure 1 (doi:10.1289/ehp.0900834.S1)]. The latter plate contained numerous triazine herbicides that did not result in any significant inhibition in any assay. Although three compounds [carbophenothion, triclosan, and triphenyl phosphate (TPP)] gave positive results with three enzymes or more, all the target enzymes were esterases.

Even if the assays are of high quality, as defined by their S/B, S/N, and *Z*′ factors (described above), false positives are bound to happen as they are dependent on the compounds tested and not on the assays. False positives are mostly due to nonspecific binding, alteration of the reporting signal (quenching of the fluorescence signal, cytotoxicity to the cells, etc.), and chemical modifications during storage of the chemicals. The purpose of the counterscreening is to eliminate false positives. To reduce nonspecific inhibition, BSA (0.1 mg/mL final concentration) was added to all buffers just before use (McGovern et al. 2002). To eliminate alteration of the reporting signal, we tested the ability of each positive hit to quench the fluorescent or luminescent signal as well as its possible cytotoxic effect. Unfortunately, it is not possible to run such controls in the primary screen format. Finally, to reduce false positives resulting from some chemical modification upon storage, we prepared a fresh solution of each positive hit just before counterscreening. Out of the 69 positive hits initially found in the library screening, individual counterscreening analysis confirmed that 39 of them are effectively positive hits (see definition above), indicating an approximately 40% false-positive rate for the primary screening. This relatively high number of false positives reflects the high concentrations used for the primary screening. A lower screening concentration will have a lower number of false positives but will significantly increase the chance of false negatives, which is not desirable. Overall, using this two-step screening method, we found that 98% of the compounds tested have no effects on the tested assays.

### Individual enzymes and receptors results

For all the positive hits selected from the library screening, we determined their individual inhibition or induction potency (IC_50_ or EC_50_) toward an enzyme or a receptor (Table 4), except for the RyR assays, which are the subject of a forthcoming study. As expected, we found that sEH was strongly inhibited by two urea-containing compounds, which are a well-established class of sEH inhibitors (Morisseau et al. 1999): siduron and triclocarban [trichlorocarbanilide (TCC)]. Although siduron uses are limited, TCC is present in numerous personal care products (Ahn et al. 2008), suggesting a large exposure risk. Animal models have shown that inhibition of the sEH affects human health by altering homeostasis, blood pressure, inflammation, and pain (Morisseau and Hammock 2008).

Inhibition of the CESs by organophosphate xenobiotics (Table 4), such as carbophenothion, parathion, phosdrin, and TPP, was expected, because such compounds are common mechanistic suicide inhibitors of serine hydrolases after activation to the oxon form (Casida and Quistad 2005). Because the CESs are only slowly reactivated, there is thus a cumulative risk. Although many organophosphate insecticides have been or are being phased out around the world, TPP continues to be used both as a plasticizer and a fire retardant in electronic components. Burning or leaching of TPP from electronic waste could result in its presence in water (Owens et al. 2007). Given the role of CES in the metabolism of ester- and amide-containing xenobiotics (Satoh and Hosokawa 2006), CES inhibition could lead to increased toxicity of xenobiotics. In general, CES inhibitors contain a carbonyl that reacts with the active-site serine to form a tetrahedral intermediate (Harada et al. 2009). Thus, the inhibition of CES1 and CES2 by triclosan, present in numerous personal care products (Ahn et al. 2008), was unexpected. To understand the mechanism of action of triclosan, we determined its kinetic constant [see Supplemental Material, Figure 2 (doi:10.1289/ehp.0900834.S1)]. We found that triclosan inhibits CES1 by a competitive mechanism and a K_I_ of 105 ± 5 nM. Although not the most potent of known CES1 inhibitors, triclosan represents a lead compound for a new class of esterase inhibitors.

PON2 was first identified as an enzyme that protects humans from environmental poisoning by organophosphate derivatives (James 2006); thus, one could expect apparent inhibition of this enzyme by organophosphates as we observed (Table 4). For carbophenothion and tributyl phosphotrithioite, this is likely due to traces of oxon impurities. Interestingly, we found that, in addition to CES1 and CES2, TPP can also significantly reduce PON2 activity. Inhibition of PON2 could lead to increased atherosclerosis and cardiovascular risk (James 2006). Taken together, exposure to TPP could affect human health through various modes of action.

For the two cytochrome P450 (CYP450) activities tested, significant inhibition was observed only for CYP450 2C9 (Table 4). 2-Methylheptyl-4,6-dinitrophenyl crotonate, the active ingredient in the fungicide dinocap, was the only very potent inhibitor of this CYP450 found. Interestingly CYP450 2C9 is involved in the production of antiinflammatory and antihypertensive epoxyeicosatrienoic acids from arachidonic acid; thus, inhibition of this CYP450 could lead to increased cardiovascular risk (Morisseau and Hammock 2008).

Screening results for the three nuclear receptor signaling pathways (AhR, ER, and AR) identified seven compounds with significant agonist activity: two for AhR, five for ER, and none for AR. Interestingly, even given the promiscuity of AhR ligand binding (Denison and Heath-Pagliuso 1998; Denison and Nagy 2003), only two fungicide chemicals, 2-(4-chlorophenyl)-benzothiazole (CPB) and dichlone, induced AhR-dependent gene expression, and they were relatively weak inducers. CPB and dichlone EC_50_ values for induction (Table 4) were approximately 5 × 10^5^-fold less potent than the prototypical AhR agonist TCDD. Although dichlone is a newly identified AhR agonist, CPB was previously reported to induce AhR-dependent expression of cytochrome CYP450 1A1 in human and mouse cell lines (Kärenlampi et al. 1989). As expected, we found that ER signal transcription was activated by *o*,*p*′-DDT (dichlorodiphenyltrichloroethane) and its metabolites *o*,*p*′-DDE (dichlorodiphenyldichloroethylene) and *o*,*p*′-DDD (dichlorodiphenyldichloroethane) (Chen et al. 1997; Rogers and Denison 2000), and our screening identified *o*,*p*′-DDE and *o*,*p*′-DDD as activators also (*o*,*p*′-DDT was not present in the screened library). In our system, the EC_50_ for induction by *o*,*p*′-DDE and *o*,*p*′-DDD was approximately 10^5^-fold less potent than that of E_2_ (Rogers and Denison 2000). Similarly, bisphenol A (BPA) and lindane have also been previously identified as ER agonists (Bonefeld-Jørgensen et al. 2007; Maranghi et al. 2007; Steinmetz et al. 1996; Vandenberg et al. 2009), although lindane has been suggested to activate ER-dependent gene expression through a nonclassical mechanism (Steinmetz et al. 1996). BPA was the most potent ER agonist identified, only 3,000-fold less potent than E_2_, whereas lindane was the weakest. Taken together, the relatively low potency of these agonists coupled with existing controversies regarding exposure and health risks associated with BPA and other endocrine-disrupting chemicals (Vandenberg et al. 2009) suggests that the adverse effects of these chemicals remain to be determined.

**Figure 1:**
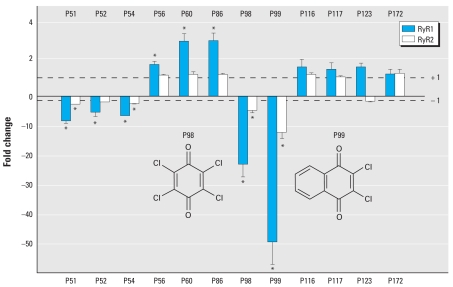
Effect of 5 μM of ferbam (P51), maneb (P52), tetramethyl-thiuram disulfide (P54), pirimiphos-methyl (P56), chlorpyrifos (P60), *o*,*p*′-DDE (P86), chloranil (P98), dichlone (P99), baythroid (P116), α-cypermethrin (P117), deltamethrin (P123), and *N*-cyclohexyl-2-benzothiazyl sulfonamide (P172) on the binding of [^3^H]Ry to the RyRs compared with DMSO control. A positive value indicates that the binding of [^3^H]Ry was increased; a negative value indicates that the binding of [^3^H]Ry was inhibited. The dashed lines at 1 and –1 are reference lines for no change in the binding of [^3^H]Ry to the receptors. *p < 0.01.

Our primary screen revealed that numerous compounds affected the RyRs, such as triclosan, which we previously showed to increase [^3^H]Ry binding to RyR1 (Ahn et al. 2008). For counterscreening, we concentrated on the 12 chemicals that produced the most significant RyR effect (Figure 1). Overall, the profiles for both receptors are similar, with the profile of RyR2 being more attenuated than that for RyR1. For the latter protein, we found eight compounds (at 5 μM) that significantly affected the binding of [^3^H]Ry: five of them inhibited the binding, and three increased it. For RyR2, we found four compounds that significantly inhibited this receptor. For both receptors, the largest effect was observed for chloranil (IC_50_ < 1.0 μM) and dichlone (IC_50_ < 1.0 μM), which both contain in their structure a 2,3-dichloro-1,4-quinone. These results are consistent with our previously published work showing that naphthoquinones and benzoquinones are capable of selectively modifying RyR1 channels in a time- and concentration-dependent manner (Feng et al. 1999). Interestingly, we found that [^3^H]Ry binding to RyR1 was increased almost 3-fold by chlorpyriphos and *o*,*p*′-DDE. Counterscreening results suggested that baythroid, α-cypermethrin, deltamethrin, and *N*-cyclohexyl-2-benzothiazyl sulfonamide have no significant effect on either RyR at 5 μM. Obtaining a compound that interacts specifically with only one of the RyRs or has opposing effects on both proteins will be scientifically very important. The deltamethrin scaffold could be a lead toward such compounds, because deltamethrin seemed to have opposing effects on both RyRs. RyR1 and RyR2 are major components of skeletal and cardiac muscle excitation contraction coupling, and several heritable mutations in these proteins have been associated with myogenic disorders (Bellinger et al. 2008). In addition, RyR1 and RyR2 are the major isoforms expressed in neurons and are responsible for producing temporally and spatially defined Ca^2+^ signals important for neuronal growth and plasticity (Berridge 2006). Deregulation of RyR function and expression contributes to alterations in activity-dependent dendritic growth and plasticity (Kenet et al. 2007; Roegge et al. 2006; Yang et al. 2009) and the balance of excitatory and inhibitory neurotransmission in the hippocampus CA1 region (Kim et al. 2009). Thus, exposure to the RyR channel activators and inhibitors identified here could trigger adverse contractile responses in muscle cells and affect proper brain development, especially in susceptible individuals.

## Conclusion

The HTS method described herein allowed the elimination of 98% of the compounds as negative hits. Furthermore, we were able to correctly identify compounds that were previously shown to inhibit or induce a particular enzymes or receptor; however, we also discovered new effects of some xenobiotics. For example, the inhibition of CES1 and CES2 by triclosan was totally unexpected, as was the inhibition of the RyRs by chloranil and dichlone. These *in vitro* results raise significant biological/toxicologic questions and further *in vivo* studies are necessary before drawing any conclusions on the health risks associated with any of these compounds by these specific mechanisms. Overall, our study shows the feasibility of using combined HTS assays as an approach toward obtaining toxicologic data on the many thousands of anthropogenic compounds for which there is little if any information. Furthermore, the HTS assays were very useful for quickly identifying compounds of potential risk for further studies, thus concentrating resources on the potentially most significant chemicals.

The National Library of Medicine has developed the infrastructure to screen compounds on possible pharmacologic leads and to report the data in an easily accessible publically available format; this is part of the National Institutes of Health Molecular Libraries Roadmap initiative. The results for the screening of sEH in this system are available online (National Center for Biotechnology Information 2009); the AhR CALUX bioassay is currently used in the same program. One useful rapid approach would be for investigators or the National Institute of Environmental Health Sciences to propose toxicologically relevant assays and also provide environmentally or industrially important compounds to the system.

## Figures and Tables

**Table 1:** Conditions for human enzyme-based bioassays.

Enzyme	Preparation used	Substrate	Concentration (μM)	Buffer	End point measured	Reference
sEH	Recombinant purified enzyme	CMNPC	5	Bis-Tris/HCl pH 7.0, 25 mM	Fluorescence kinetic	Jones et al. 2005
CES1	Recombinant partially purified enzyme	CMNA	50	Na_2_PO_4_ pH 7.4, 0.1 M	Fluorescence kinetic	Shan and Hammock 2001
CES2	Recombinant partially purified enzyme	CMNA	50	Na_2_PO_4_ pH 7.4, 0.1 M	Fluorescence kinetic	Shan and Hammock 2001
CES3	Recombinant partially purified enzyme	CMNA	50	Na_2_PO_4_ pH 7.4, 0.1 M	Fluorescence kinetic	Shan and Hammock 2001
FAAH	Recombinant microsomes	Octanoyl-MP	50	Na_2_PO_4_ pH 8.0, 0.1 M	Fluorescence kinetic	Huang et al. 2007
PON2	Recombinant microsomes	CMNA	50	Na_2_PO_4_ pH 7.4, 0.1 M	Fluorescence kinetic	Shan and Hammock 2001
GSTs	Pooled human liver cytosol	CDNB	1,000	K_2_PO_4_ pH 6.5, 0.1 M	Absorbance kinetic	Habig et al. 1974
CYP450 1A2 and 2C6	Pooled human liver microsomes	EROD	25	K_2_PO_4_ pH 7.4, 0.1 M	Fluorescence kinetic	Dutton et al. 1989
CYP450 2C9	Pooled human liver microsomes	Luciferin H	50	K_2_PO_4_ pH 7.4, 0.1 M	Luminescence	Cali et al. 2006

Abbreviations: CDNB, 1-chloro-2,4-dinitrobenzene; CMNA, cyano(6-methoxy-2-naphthyl)methyl actetate; CMNPC, cyano(6-methoxy-naphthalen-2-yl)methyl *trans*-[3-phenyloxiran-2-yl)methyl] carbonate; EROD, ethoxyresorufin; Octanoyl-MP, *N*-(6-methoxypyridin-3-yl) octanamide.

**Table 2:** Conditions for cell-based bioassays.

Human receptor	Acronym	Preparation used	Substrate	End point measured	Reference
Aryl hydrocarbon receptor	AhR	Recombinant cells	Luciferin	Luminescence	Han et al. 2004
Androgen receptor	AR	Recombinant cells	Luciferin	Luminescence	Rogers and Denison 2000
Estrogen receptor	ER	Recombinant cells	Luciferin	Luminescence	Rogers and Denison 2000
Ryanodine receptor 1	RyR1	Skeletal muscle membranes	[^3^H]Ry	Radioactivity	Pessah et al. 1987
Ryanodine receptor 2	RyR2	Ventricular muscle membranes	[^3^H]Ry	Radioactivity	Pessah et al. 1990

**Table 3:** Characteristics and positive primary screen results for enzyme- and cell-based bioassays.

	Assay characteristics			No. of positive results	
Assay	S/B^a^	S/N^a^	*Z*′^a^	Primary screen	Counterscreen
Enzyme					
sEH	4.0 ± 0.1	38 ± 8	0.8 ± 0.1	2	2
CES1	11 ± 3	19 ± 2	0.8 ± 0.1	4	2
CES2	9.2 ± 0.9	106 ± 33	0.8 ± 0.1	4	3
CES3	6.1 ± 0.7	28 ± 7	0.7 ± 0.1	7	4
FAAH	150 ± 10	35 ± 5	0.8 ± 0.1	0	—
PON2	18 ± 2	134 ± 31	0.8 ± 0.04	4	3
GSTs	2.4 ± 0.5	28 ± 9	0.7 ± 0.05	0	—
CYP450 1A2 and 2C6	13 ± 5	48 ± 10	0.7 ± 0.1	1	0
CYP450 2C9	19 ± 4	79 ± 18	0.7 ± 0.05	12	7
Receptor					
AhR	32 ± 1	410 ± 30	0.6 ± 0.2	3	2
AR	18 ± 2	180 ± 70	0.7 ± 0.1	0	—
ER	5 ± 1	80 ± 40	0.6 ± 0.1	8	5
RyR1	170 ± 30	500 ± 90	0.5 ± 0.1	12	8
RyR2	100 ± 10	310 ± 40	0.6 ± 0.1	12	4

a Results are mean ± SD of at least four independent measurements.

**Table 4:** Positive counterscreen results for the enzyme assays and nuclear receptor–based bioassays.

Assay	Compound	IC_50_ or EC_50_ (nM)^a^	Use
sEH	Siduron	33 ± 3	Herbicide
	TCC	13 ± 1	Microbiocide

CES1	Triclosan	210 ± 20	Microbiocide
	TPP	43 ± 3	Flame retardant

CES2	Carbophenothion	34 ± 1	Insecticide
	Triclosan	580 ± 30	Microbiocide
	TPP	50 ± 2	Flame retardant

CES3	Carbophenothion	110 ± 15	Insecticide
	Parathion	4.9 ± 0.4	Insecticide
	Phosdrin	1.1 ± 0.1	Insecticide
	Primiphos-ethyl	180 ± 20	Insecticide

PON2	Carbophenothion	110 ± 6	Insecticide
	Tributyl phosphorotrithioite	120 ± 10	Herbicide
	TPP	85 ± 8	Flame retardant

CYP450 2C9	2-Butan-2-yl-4,6-dinitro-phenol	1,900 ± 100	Pesticide
	Chlorpyrifos	3,200 ± 200	Insecticide
	Finasteride	1,500 ± 100	Antiandrogen
	2-Methylheptyl-4,6-dinitrophenyl crotonate	120 ± 1	Fungicide
	Pentachlorophenol	850 ± 10	Herbicide
	Pyrethrum	2,300 ±100	Insecticide
	Triclosan	650 ± 40	Microbiocide

AhR	CPB	11,400	Fungicide
	Dichlone	> 10,000	Fungicide

ER	BPA	330	Plastic monomer
	*o*,*p-*DDD (dichlorodiphenyldichloroethane)	1,200	Insecticide
	*o*,*p-*DDE (dichlorodiphenyldichloroethylene)	1,200	Insecticide
	Endrin	13,000	Pesticide
	Lindane	> 50,000	Insecticide

a Values are IC_50_s for the enzyme-based assays (sEH to CYP450 2C9) and EC_50_s for the receptor-based assays (AhR and ER). Results are mean ± SD of at least three independent measurements.
